# Graph Autoencoder with Preserving Node Attribute Similarity

**DOI:** 10.3390/e25040567

**Published:** 2023-03-26

**Authors:** Mugang Lin, Kunhui Wen, Xuanying Zhu, Huihuang Zhao, Xianfang Sun

**Affiliations:** 1 College of Computer Science and Technology, Hengyang Normal University, Hengyang 421002, China; 2 Hunan Provincial Key Laboratory of Intelligent Information Processing and Application, Hengyang 421002, China; 3 School of Computer Science and Informatics, Cardiff University, Cardiff CF24 4AG, UK

**Keywords:** graph representation learning, graph autoencoder, unsupervised learning, *k*-nearest neighbor

## Abstract

The graph autoencoder (GAE) is a powerful graph representation learning tool in an unsupervised learning manner for graph data. However, most existing GAE-based methods typically focus on preserving the graph topological structure by reconstructing the adjacency matrix while ignoring the preservation of the attribute information of nodes. Thus, the node attributes cannot be fully learned and the ability of the GAE to learn higher-quality representations is weakened. To address the issue, this paper proposes a novel GAE model that preserves node attribute similarity. The structural graph and the attribute neighbor graph, which is constructed based on the attribute similarity between nodes, are integrated as the encoder input using an effective fusion strategy. In the encoder, the attributes of the nodes can be aggregated both in their structural neighborhood and by their attribute similarity in their attribute neighborhood. This allows performing the fusion of the structural and node attribute information in the node representation by sharing the same encoder. In the decoder module, the adjacency matrix and the attribute similarity matrix of the nodes are reconstructed using dual decoders. The cross-entropy loss of the reconstructed adjacency matrix and the mean-squared error loss of the reconstructed node attribute similarity matrix are used to update the model parameters and ensure that the node representation preserves the original structural and node attribute similarity information. Extensive experiments on three citation networks show that the proposed method outperforms state-of-the-art algorithms in link prediction and node clustering tasks.

## 1. Introduction

Graphs are an essential tool to describe and model various complex systems in the real world, where their nodes can represent the entities in complex systems, and their edges can effectively describe the relationship between entities. For instance, the social network between individuals formed by QQ, WeChat, and Weibo, the network of web links consisting of thousands of pages on the Internet, and the logistics network consisting of transport traffic between cities, all can be modeled as graphs. Moreover, many real-world problems can be solved by transforming them into optimization problems on graphs. For example, identifying hackers or terrorists can be considered as a problem of detecting anomalous nodes in a graph, and knowledge graph completion can be viewed as a link prediction problem in a graph. Therefore, graph deep learning has recently emerged to solve many problems in graphs and tasks of graph analysis. However, in contrast to regular Euclidean space data, graphs have a nonlinear data structure defined in an irregular non-Euclidean space. This nonlinearity is due to the disorder of nodes and the connectivity between them. An adjacency matrix is a straightforward representation of a graph. However, this representation only captures neighboring relationships between vertices and cannot describe higher-order structure information, such as paths. Furthermore, for large-scale graphs, adjacency matrices have high dimensionality and data sparsity. Therefore, traditional deep learning techniques designed for regularly structured data cannot be applied to graph-structured data. Transformation of graph-structured data into general data that traditional methods can easily process is a challenging task in deep learning.

Graph representation learning is effective for obtaining latent low-dimensional representations that facilitate subsequent graph analysis tasks by learning graph structure and node attributes, while ultimately preserving the properties required for subsequent tasks [1,2]. In recent years, graph representation learning has been widely studied, and several graph representation learning methods have been proposed. For instance, the matrix factorization-based methods obtain low-dimensional representations by factorizing the adjacency matrix using suitable methods such as GraRep [3], HOPE [4], and M-NMF [5]. Random walk-based methods, such as DeepWalk [6], Node2vec [7], and Metapath2vec [8], learn local neighborhood connectivity and global structure information by traversing a graph to obtain its low-dimensional representation. Proximity-based methods, such as SDNE [9] and LINE [10], apply deep learning methods that use proximity loss functions to preserve the node proximity in a graph, such that nodes that are close together in the input graph are likewise in the embedding space. In the real world, nodes in most graphs come with rich attribute information. However, while most proposed methods design algorithms based solely on the topological structure of graphs, they often overlook valuable attribute information associated with nodes. In reality, node attributes and graph structure are two distinct, yet complementary sets of information for successful graph analysis tasks in attribute networks. Therefore, for effective graph analysis in attribute networks, both the topological structure of networks and the attribute information associated with nodes must be considered.

In recent years, graph convolutional networks (GCNs) have demonstrated high performance in graph representation learning due to their capacity to aggregate and transform information from graph structure and node attributes within a node’s neighbors into node representations [11,12,13,14,15]. GCN is a semi-supervised graph representation learning method that requires a wide range of labeled nodes for representation learning. Since labeling nodes is usually expensive, and the graph representation learning is considered a generic task independent of the downstream tasks, graph representation learning is typically performed in an unsupervised learning manner. Graph AutoEncoders (GAEs) [12], which are unsupervised learning models composed of the concept of autoencoders with GCN and graph representation learning, have been researched lately. GAEs consist of two parts: an encoder and a decoder. The encoder uses GCNs to embed the nodes into a low-dimensional space by learning from the adjacency matrix and node attribute matrix of an input graph. The decoder reconstructs the adjacency matrix of the graph by a simple inner product. During network training, the network preserves the topological structure of the graph by the decoder to perform unsupervised learning. Many GAE variants have been proposed to learn node representation in an unsupervised way. Due to their high node representation ability under unsupervised learning, they have been utilized in various graph analysis tasks, such as node clustering [16,17], link prediction [18,19], and graph anomaly detection [20,21].

Although many GAE-based methods have been proposed, unsupervised learning of these models is highly dependent on the reconstruction of the graph structure and ignores the attribute information of nodes. Therefore, these models cannot determine the amount of information on the latent node attributes contained in the node embedding. Consequently, these models do not ensure that the node vector representation contains reliable node attribute information. Additionally, most GAE models use the GCN as their encoders, and a fixed 0/1 adjacency matrix and a node attribute matrix as input. During model training, when aggregating node attributes, each neighbor node is aggregated using the same weight. Thus, the aggregation process of GCN smooths the node attributes, preserving the structural proximity, while destroying the node attribute similarity of the original attribute space [22]. The node attribute similarity plays a crucial role in many graph analysis tasks. For instance, in attribute networks, node clustering consists of partitioning the nodes based on attribute and structure similarities, and link prediction consists of predicting whether an edge exists between every two nodes based on these similarities. For graph representation learning, the node information is mapped from an original high-dimensional space to a latent low-dimensional space. Furthermore, regardless of the different node representations between the original space and the latent space, it is expected that the similarity between the nodes in the two spaces is preserved. This is particularly desirable for downstream tasks, such as node clustering and link prediction.

In this paper, we aim to propose a novel graph autoencoder model that can better maintain the attribute similarity between nodes. Two main challenges were faced when designing this model. Firstly, the designed GAE framework should effectively fuse topological structure and node attributes to alleviate the destruction of node attribute similarity by neighbor smoothing during the aggregation process of GCN. To address this, a fusion strategy that naturally incorporates the original graph structure and the node attribute neighbor graph based on node attribute similarities as the input of the graph convolutional encoder was proposed in the designed GAE framework. In the graph convolutional encoder, the node aggregation operation can be decomposed into two parts: the mean aggregation from node neighbors in the original graph and the linear aggregation by similarity coefficients from node attribute neighbors. The aggregation process alleviates the destruction of node attribute similarity and strengthens its maintenance. Secondly, the node attribute similarity should be preserved in an unsupervised learning manner. Thus, when developing the structure reconstruction decoder, a similarity reconstruction decoder that directly computes the similarity between node representation vectors and steers the unsupervised learning of the model by the node similarity difference between the original attribute space and in embedding space is added. As a result, the model ensures that the node representations preserve both the structure and attribute similarities between nodes.

The main contributions of this paper can be summarized as follows:

(1) A novel GAE framework integrating node attribute similarity and structural information is proposed. This new framework can effectively reconstruct node attribute similarity, ensuring that the obtained node representations maintain the same node attribute similarity between nodes as the original attribute space.

(2) An effective fusion strategy for node attribute similarity and structural information is proposed. An attribute neighbor graph is constructed by using the attribute similarity between nodes as edge weights, and then integrated with the original graph as the structural input of the GCN encoder. In this way, nodes can aggregate their neighbor attribute information in two distinct ways in the GCN encoder to effectively fuse node attribute similarity and structural information while avoiding over-smoothing of GCN to some extent.

(3) Extensive experiments on link prediction and node clustering are conducted on three real-world datasets to evaluate the effectiveness of the proposed model. The experimental results show that the proposed model outperforms state-of-the-art methods in two graph analysis tasks.

## 2. Preliminary and Related Work

In this section, the notations and definitions used in this paper are introduced, and the attributed graph representation learning and GAE models in attribute graphs are then reviewed.

### 2.1. Notations and Definitions

**Definition** **1** *(Graph). A graph is defined as *G=(V,E)*, where *$V=\{v_{1},v_{2},\cdots ,v_{n}\}$ *denotes a set of nodes with *$\left|V\right|=n$ *and* E⊆V×V *representing a set of edges connecting node pairs.*

The graphs are usually represented by an adjacency matrix $A=[a_{ij}]\in R^{n\times n}$. For an unweighted graph, if there exists an edge $e_{ij}$ between nodes $v_{i}$ and $v_{j}$, then $a_{ij}=1$. Otherwise, $a_{ij}=0$. For a weighted graph, $a_{ij}$ is a non-negative weight associated with the edge $e_{ij}$. If $v_{i}$ and $v_{j}$ are not directly connected, then $a_{ij}=0$.

**Definition** **2** *(Attributed graph). An attributed graph is defined as a graph* G=(V,E,X)*, where* V *is a set of nodes with* $\left|V\right|=n$ *and* E⊆V×V *representing a set of edges in graph* G. $X=[X_{1},X_{2},\cdots ,X_{n}]\in R^{n\times c}$ *is the node attribute matrix, where* $X_{i}\in R^{n}$ *represents the attributes associated with node* $v_{i}$*,* c *is the dimension of the node attributes, and* $x_{ij}$ *is the value of the* i*-th node on the* j*-th attribute.*

**Definition** **3** *(Graph representation learning). Given an attribute graph G = (V, E, X), the task of graph representation learning is to learn a mapping function $f:v_{i}\to z_{i}\in R^{d}$, where z_i_ is the latent representation of node v_i_ in a d dimensional latent vector space. The transformation f should preserve the original graph information so that two similar nodes in the original graph are represented similarly in the latent vector space.*

Graph representation learning, also known as network embedding, aims to map graph data into a latent space making it more convenient to cope with subsequent tasks. Therefore, the node representations should satisfy the following conditions: (1) To improve the computational efficiency of subsequent tasks, the dimension of the node representation vectors should be much smaller than the number of nodes in the network, i.e., d≪|V|. (2) The node representation vectors should have continuous real values to facilitate subsequent tasks using classical methods. (3) The node representation vectors should preserve the node similarity as reflected by node attributes and graph structure in the original graph. In graph representation learning, two similar nodes in the original graph should preserve the similarity in the embedded representation space.

For an attribute graph, the node similarity is generally measured using structural proximity and attribute similarity.

**Definition** **4** *(Structure proximity). The structure proximity is the similarity of graph structure information between nodes, which plays an important role in preserving the graph structure information in graph representation learning. Three different measures of structure proximity exist. The first-order proximity describes the local pairwise proximity between nodes linked by edges. For two nodes* $v_{i}$ *and* $v_{j}$*, if they have an edge directly connecting them, then the first-order proximity between them is equal to the edge weight; otherwise, it is null. The first-order proximity matrix is the adjacency matrix. The second-order and high-order proximities describe the similarities of second-order and higher-order neighbors between nodes, respectively. For two nodes* $v_{i}$ *and* $v_{j}$*, the second-order proximity is determined by the number of common neighbors of the two nodes, and the high-order proximity is measured by calculating the* k*-step transition probability from* $v_{i}$ *to* $v_{j}$.

In graph representation learning, it is necessary to preserve the first-order proximity. This is because if two nodes are connected by an edge with greater weight, they are more similar in the real world and should be closer to each other in the embedding space. For example, self-supervised learning of GAE is performed by preserving the first-order proximity of graphs [12]. In this paper, only the first-order proximity of nodes is considered.

**Definition** **5** *(Attribute similarity). The attribute similarity describes the similarity of attribute information between nodes. Given an attribute graph* G=(V,E,X)*, the attribute similarity between nodes* $v_{i}$ *and* $v_{j}$ *is computed by the attribute vector* $X_{i}$ *of node* $v_{i}$ *and the attribute vector* $X_{j}$ *of node* $v_{j}$.

### 2.2. Attributed Graph Representation Learning

The early methods for graph representation learning focus on the graph’s structural information. These methods only consider the graph structural information to learn node representation while ignoring the node attribute information, and thus their performance is not satisfactory [1,2,3,4,5,6,7,8,9,10]. In attribute graphs, node attributes can provide an effective complement to graph structural information. Nevertheless, node attributes and graph structure are two distinct types of information, making effective use of node attributes for graph representation learning a challenging task. In graph representation learning, there are three ways to integrate node attributes with graph structure information into the node representation. The straightforward approach involves first learning representation from node attributes and graph structure information separately, and then concatenating or fusing the two individual representations. However, since both types of information are relevant, the learning process must take into account their interaction. For instance, DANE [23] uses two autoencoders to learn the structural and node attribute information, and then concatenates the two representations together as a node representation. By introducing the corresponding loss functions, the node representation is guaranteed to possess consistent and complementary information from the graph structure and node attributes. RolEANE [24] uses two autoencoders to learn the structural and node attribute information separately, and subsequently applies a neighbor-modified skip-gram model integrating the two kinds of information. To effectively capture the important potential information about complex coupling and interaction in the network, the model adopts two structural role proximity enhancement strategies that improve the structural role of proximity. The second way consists of fusing the node attribute and graph structure information before performing representation learning. Although the two types of information are learned together, effectively fusing them can be challenging. For instance, ASNE [25] first integrates structural and attribute information, and then uses a neural network to learn its representation. NetVAE [26] uses a shared encoder to learn node representation from graph structure, as well as node attribute information, and a dual decoder to separately reconstruct the two types of information. The third way consists of integrating the two types of information—graph structure and node attributes—using one to constrain the operation of the other. TADW [27] is proposed as a matrix factorization framework incorporating DeepWalk and contextual text features. It uses the textual feature matrix of nodes to restrict the matrix decomposition process, which combines the structural information with textual features to compensate for the lack of structural information in the network. However, it is a linear model and is insufficient for a sophisticated attribute network. Graph neural networks (GNNs) have recently become an important tool for graph representation learning. GNNs [11,28,29,30,31] mostly follow a recursive neighborhood scheme, using graph structural information to constrain each node to aggregate the attribute vectors of its neighborhood, and thus update its feature vector. After *k* aggregation iterations, a node representation is obtained, which fuses the structural and node attribute information within the node’s *k*-hop neighborhood.

### 2.3. GAE Models in Attribute Graphs

Preserving graph topological information, which is the fundamental information of graph-structured data, is crucial for graph representation learning. In addition to the structural information, nodes in attribute networks also have rich attribute content attached to them, which significantly affects the formation of networks and is an effective complement to graph structural information. Thus, it is expected to preserve the corresponding node attribute information. However, most GAE models only reconstruct the graph structure in unsupervised learning, which means that they do not ensure reliable node attribute information contained in the node representation. To overcome this limitation, Park et al. [32] proposed the symmetric graph convolutional autoencoder which can use both the graph structure and the node attribute information throughout the entire encoding–decoding process. However, its decoder reconstructs the node attribute matrix instead of the adjacency matrix of the graph, making it impractical. Recently, some methods have used two decoders to separately reconstruct the graph structure and node attributes from low-dimensional node representations, to ensure that the node representation can preserve the structural information of the input graph and the attribute information of the nodes. Zhou et al. [33] and Sun et al. [34] proposed two deep learning frameworks to obtain a latent embedding by integrating both the structure information and attribute information in a GAE with dual decoders, where one inner product decoder is used to reconstruct the graph adjacency matrix, and the other graph convolutional decoder is used to reconstruct the node attribute matrix. Similarly, Wang et al. [35] designed a novel GAE with two decoders, called GASN, where the encoder is a low-pass graph filter, one decoder uses a high-pass graph filter to reconstruct node attributes, and the other is an inner product decoder. Although these GAE models adopt two decoders to respectively reconstruct the structure and attribute features of nodes, the effectiveness of learned node representations is reduced because they use graph convolutional neural networks as their encoders, which to some extent destroy the attribute similarity between nodes in the original space during the aggregation process [22]. The main differences between these models above and our proposed model are as follows: (1) Our model reconstructs the structure and attribute similarity between nodes with two decoders, whereas the models above reconstruct the structure and node attributes. (2) Our model uses two different aggregation methods in the GCN encoder aggregation process to aggregate neighborhood attributes in the original structure graph and attribute similarity neighborhood graph, respectively, effectively eliminating the destruction of attribute similarity between nodes in the original space by GCN, whereas the models above use the common GCN mean aggregation method in the GCN encoder aggregation process, which destroys to some extent attribute similarity between nodes in the original space. Currently, some GAE methods are studied from multi-scale and multi-view perspectives. Guo et al. [19] proposed a novel multi-scale variation GAE to improve the robustness of existing GAE models. The model learns the adjacency matrix and attribute matrix with different scales by the encoder, and reconstructs the two matrices by the decoder to preserve the original structural and attribute information. MVGAE [36] is a multi-view GAE that can aggregate latent information from local topology, global topology, and feature similarity. It uses an attention mechanism to fuse the three kinds of information into node representation and simultaneously reconstruct the graph structure and node features. Similarly, A2AE [37] first embeds the attribute multi-view graph into latent representations by multi-encoders, then fuses the view-specific latent representations into node representation using the attention mechanism, and finally reconstructs the graph structure and attributes using multi-decoders.

## 3. Graph Autoencoder with Preserving Node Attribute Similarity

In this section, the graph autoencoder with preserving node attribute similarity (GAEPNAS) is presented, including the basic model framework, fusion module with graph structure and node attribute similarity, encoder module, decoder module, and training algorithm.

### 3.1. Overall Model Framework

**Figure 1:**
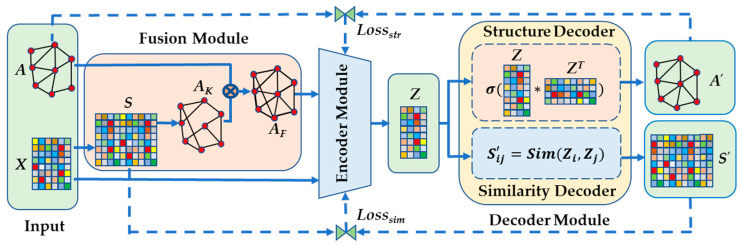
Overall Framework of the proposed GAEPNAS model.

Given an attribute graph G=(V,E,X), the topology structure of graph G is designated by an adjacency matrix A and X denotes an attribute matrix of the nodes. The objective is to embed graph structures and node attributes into a low-dimensional representation Z using GAEPNAS, where Z preserves the attribute similarity of the nodes in X and the graph structural information. The overall framework of the proposed GAEPNAS model is shown in Figure 1.

The model is mainly composed of three modules: a fusion module, an encoder module, and a decoder module. First, in the fusion module, a *k*-nearest-neighbor (KNN) graph is constructed based on the similarities between nodes. The KNN graph and the original input graph are then integrated into a synthetic graph to combine the graph structure information and node attribute similarity information in a unified way. Second, in the encoder module, the latent node representation is obtained through *l*-layers GCN to learn from the synthetic graph. Third, in the decoder module, dual decoders are used to reconstruct the graph adjacency matrix and the node attribute similarity matrix. Finally, the loss function containing a structural loss and a loss of node attribute similarity is calculated to update the parameters in reverse and thus ensure that the node representation preserves the original structural and node attribute similarity information.

### 3.2. Fusion Module

In attribute networks, similarities exist between the nodes in terms of both the network structure space and the node attribute space. However, it is important to note that just because two nodes have a similar network structure does not necessarily mean they have similar node attributes and vice versa. Thus, in graph representation learning, it is crucial to consider the dependency of both spaces and align them cohesively. To learn the node attribute similarity information using GAE, the attribute similarity relationship between nodes must be converted into an attribute neighbor space. Constructing KNN graphs is an effective way to achieve this conversion. Therefore, a KNN graph is first constructed based on the similarities between nodes. The KNN graph and the input graph are then integrated into a synthetic graph so that the attribute similarity information can be fused with the structural information. The process is described below.

(1) Calculating the attribute similarity matrix

The attribute similarity matrix $S=[S_{ij}]\in R^{n\times n}$ where $S_{ij}$ represents the attribute similarity between nodes *v_i_* and *v_j_*, is calculated by a similarity function $Sim(X_{i},X_{j})$ on attributes *X_i_* and *X_j_* as

(1) $$S_{ij}=Sim\left(X_{i},X_{j}\right)$$

This similarity function can be a cosine similarity function $Cos\left(X_{i},X_{j}\right)=\frac{X_{i}X_{j}^{T}}{\left\|X_{i}\right\|\left\|X_{j}\right\|}$ or other similarity functions such as Jaccard coefficient, Euclidean distance, and Pearson correlation, depending on the characteristics of the node attributes.

(2) Constructing a KNN graph

In the attribute similarity matrix S, if the value of $S_{ij}$ is closer to 1, $X_{i}$ and $X_{j}$ are more similar, which indicates that it is more likely that the two nodes $v_{i}$ and $v_{j}$ are linked. Thus, given a parameter k, a KNN graph $G_{K}$, based on the similarity between each node pair in the similarity matrix S, is generated. The node set of the graph $G_{K}$ is similar to that of the input graph. If $X_{j}$ is one of the top-k similar neighbors of $X_{i}$, or the latter is one of the top-k similar neighbors of $X_{j}$, then an edge between $v_{i}$ and $v_{j}$ in the KNN graph exists. Otherwise, there is no edge between $v_{i}$ and $v_{j}$. The weight of the edge connecting nodes $v_{i}$ and $v_{j}$ is calculated as

(2) $$\begin{array}{c} A_{K_{ij}}=\left\{\begin{array}{c} S_{ij},X_{j}\in N_{k}\left(X_{i}\right)⋁X_{i}\in N_{k}\left(X_{j}\right)\\ 0,X_{j}\notin N_{k}\left(X_{i}\right)⋀X_{i}\notin N_{k}\left(X_{j}\right) \end{array}\right. \end{array}$$

where $N_{k}(X_{i})$ denotes the node set of the top-k similar neighbors of $X_{i}$.

For $X_{j}\notin N_{k}\left(X_{i}\right)⋀X_{i}\notin N_{k}\left(X_{j}\right)$, it is considered that $X_{i}$ and $X_{j}$ are dissimilar, and thus $A_{K_{ij}}=0$. If $X_{j}\in N_{k}\left(X_{i}\right)⋁X_{i}\in N_{k}\left(X_{j}\right)$, $X_{i}$ and $X_{j}$ are more similar, and the weight $A_{K_{ij}}$ of the edge linking the two nodes $v_{i}$ and $v_{j}$ is greater. Note that if $A_{K_{ii}}=1$, node $v_{i}$ has a self-loop, which is not currently considered and will be considered again in the encoder module, and therefore it can be assumed that $A_{K_{ii}}=0$. Therefore, the adjacency matrix $A_{K}$ is given by

(3) $$A_{K}=\left[\begin{array}{cccc} 0 & A_{K_{12}} & \cdots & A_{K_{1n}}\\ A_{K_{21}} & 0 & \cdots & A_{K_{2n}}\\ \vdots & \vdots & \ddots & \vdots \\ A_{K_{n1}} & A_{K_{n2}} & \cdots & 0 \end{array}\right]$$

(3) Integrating KNN graph with original input graph

For attributed networks, the structural and node attribute information are two different types of information that are independently and mutually complementary. Effectively integrating them is a challenging task. In this paper, the KNN graph $G_{K}$ and the original input graph G are integrated into a synthetic graph $G_{F}$ with edge weights. The adjacency matrix $A_{F}$ of the graph $G_{F}$ is calculated as

(4) $$A_{F}=\beta A+(1-\beta )A_{K}$$

where β∈[0,1] is a parameter that balances the effect of the input graph and the KNN graph.

Analyzing matrix $A_{F}$ using Equation (4) shows that matrix $A_{F}=[A_{F_{ij}}]\in R^{n\times n}$ is a symmetric matrix and $A_{F_{ij}}\in [0,1]$ i,j∈{1,2,…,n} The pseudocode of the whole fusion module is summarized in Algorithm 1.
**Algorithm 1.** Fusiongraph**Input:** Adjacency matrix A, Attribute matrix X, Number of nearest neighbor k, and β.**Output:** Similarity matrix $S=[S_{ij}]$ and Adjacency matrix $A_{F}=[A_{F_{ij}}]$ of graph $G_{F}$.1.**for** i=1:n      //n represents the number of nodes.2.  **for** j=i:n
3.     $S_{ij}=S_{ji}=Sim\left(X_{i},X_{j}\right)$;4.   **end**5.**end**6.Let $A_{K}=[A_{K_{ij}}]={\left[0\right]}_{n\times n}$; //$A_{K}$ denotes the adjacency matrix of the KNN graph.7.**for** i=1:n8.   **for** j=1:n
9.     **if** $X_{j}\in N_{k}\left(X_{i}\right)$
**then** $A_{K_{ij}}=A_{K_{ji}}=S_{ij}$;10.   **end**11.   $A_{K_{ii}}=0$;12.**end**13.$A_{F}=\beta A+\left(1-\beta \right)A_{K}$;14.**return** S and $A_{F}$;

### 3.3. Encoder Module

The encoder is the core module of the GAEPNAS, which obtains a latent node representation by learning the attribute and structure information of the synthetic graph. GCN is an excellent graph neural network, which generalizes the convolutional neural network (CNN) in graph space and uses the first-order approximation of Chebyshev polynomials to simplify the computation [11]. Because of the powerful graph representation learning capability of GCN, GAE usually uses GCN as its encoder. In the encoder module, the graph encoder consists of l GCN convolutional layers. The input of the encoder is the adjacency matrix $A_{F}$ and the attribute matrix X, while the output is the node representation matrix Z. $I_{n}$ is the n×n identity matrix. $\tilde{A}=I_{n}+A_{F}$ (by adding a self-loop for each node, it is ensured that it also participates in the attribute aggregation of new node embedding), and the diagonal matrix $\tilde{D}$ with ${\tilde{D}}_{ii}=\sum_{j=1}^{n}{\tilde{A}}_{ij}$, are first calculated. The l-layer GCN encoder model learns the node representation with the following propagation rule:

(5) $$\begin{array}{c} \begin{array}{c} H^{\left(i+1\right)}=ReLU\left({\tilde{D}}^{-\frac{1}{2}}\tilde{A}{\tilde{D}}^{-\frac{1}{2}}H^{\left(i\right)}W^{\left(i\right)}\right),i=0,1,\ldots \\ Z={{\tilde{D}}^{-\frac{1}{2}}\tilde{A}{\tilde{D}}^{-\frac{1}{2}}H}^{\left(l-1\right)}W^{\left(l-1\right)} \end{array} \end{array}$$

where $W^{\left(i\right)}$ is the trainable weight matrices of the i-th layer, $H^{\left(0\right)}=X$, $ReLU\left(.\right)=max(0,.)$ is the activation function for the first l−1 layers, and a linear activation function is used for the last layer. The encoder embeds the topological structure and node attribute of the input graph into representation Z.

By substituting Equation (4) and $\tilde{A}=I_{n}+A_{F}$ into Equation (5), the following is obtained:

(6) $$\begin{array}{c} \begin{array}{c} H^{\left(i+1\right)}=ReLU\left(\beta {\tilde{D}}^{-\frac{1}{2}}\left(I_{n}+A\right){\tilde{D}}^{-\frac{1}{2}}H^{\left(i\right)}W^{\left(i\right)}+(1-\beta ){\tilde{D}}^{-\frac{1}{2}}(I_{n}+A_{F}){\tilde{D}}^{-\frac{1}{2}}H^{\left(i\right)}W^{\left(i\right)}\right)\\ Z=\beta {\tilde{D}}^{-\frac{1}{2}}\left(I_{n}+A\right){\tilde{D}}^{-\frac{1}{2}}H^{\left(l-1\right)}W^{\left(l-1\right)}+(1-\beta ){\tilde{D}}^{-\frac{1}{2}}(I_{n}+A_{F}){\tilde{D}}^{-\frac{1}{2}}H^{\left(l-1\right)}W^{\left(l-1\right)} \end{array} \end{array}$$

Let $H^{\left(i\right)}=\{H_{1}^{\left(i\right)},H_{2}^{\left(i\right)},\cdots ,H_{n}^{\left(i\right)}\}$ and $Z=\{Z_{1},Z_{2},\cdots ,Z_{n}\}$$N_{Aj}$ and $N_{A_{F}j}$ are the neighbor set for node j in graph G and $G_{F}$ which contains node j, respectively. Then Equation (6) can be written in the following form:

(7) $$\begin{array}{c} \begin{array}{c} H_{j}^{\left(i+1\right)}=ReLU\left(\sum_{k\in N_{Aj}}\frac{{\beta W}^{\left(i\right)}H_{k}^{\left(i\right)}}{\sqrt{{{\tilde{D}}_{jj}\tilde{D}}_{kk}}}+\sum_{f\in N_{A_{F}j}}\frac{{\left(1-\beta \right)S_{jf}W}^{\left(i\right)}H_{f}^{\left(i\right)}}{\sqrt{{{\tilde{D}}_{jj}\tilde{D}}_{ff}}}\right)\\ Z_{j}=\sum_{k\in N_{Aj}}\frac{{\beta W}^{\left(l-1\right)}H_{k}^{\left(l-1\right)}}{\sqrt{{{\tilde{D}}_{jj}\tilde{D}}_{kk}}}+\sum_{f\in N_{A_{F}j}}\frac{{\left(1-\beta \right)S_{jf}W}^{\left(l-1\right)}H_{f}^{\left(l-1\right)}}{\sqrt{{{\tilde{D}}_{jj}\tilde{D}}_{ff}}} \end{array} \end{array}$$

It can be deduced from Equation (7) that the graph convolutional operation of the encoder can be decomposed into two convolutional suboperations: one on the original input graph and one on the KNN graph, with parameter weight matrices $W^{\left(i\right)}$ shared in the i-th layer. For the convolutional operation on the original graph, the message propagation occurs in terms of the neighbors of nodes in graph structure space, while its aggregation is mean due to the adjacency matrix A being a 0/1 matrix. In contrast, for the convolutional operation on the KNN graph, the propagation of node representation is in terms of the attribute neighbors of nodes in attribute space, and it aggregates the representation of the top-k nearest neighbors based on the different attribute similarities between the nodes. This can be interpreted as a similarity attention coefficient. Therefore, the encoder can propagate and aggregate node characteristics in both the structure space and attribute space, using sharing parameter matrices. This enables the node representation to effectively fuse information from both spaces, to alleviate the destruction of node attribute similarity, and to avoid over-smoothing of GCN to some extent.

### 3.4. Decoder Module

The decoder module consists of two decoders: a structure decoder and a similarity decoder. The structure decoder is used to reconstruct the adjacency matrix $A^{\prime }$ by computing the inner product of the node representation as

(8) $$\begin{array}{c} A^{\prime }=sigmoid\left(ZZ^{T}\right) \end{array}$$

where $sigmoid\left(x\right)=\frac{1}{1+e^{-x}}$ denotes the activation function.

The similarity decoder is used to reconstruct the attribute similarity matrix $S^{\prime }=[S_{ij}^{\prime }]\in R^{n\times n}$ between nodes in the embedded space by computing the node representation similarity between them:

(9) $$\begin{array}{c} S_{ij}^{\prime }=Sim\left(Z_{i},Z_{j}\right) \end{array}$$

where $Sim\left(.,.\right)$ is a similarity function, and $Z_{i}$ and $Z_{j}$ are node representation vectors of nodes $v_{i}$ and $v_{j}$, respectively.

The two decoders are designed to perform unsupervised learning of the GAEPNAS model by comparing the adjacency matrix and attribute similarity matrix of the embedded space with those of the original space, so that the whole encoding–decoding process can make full use of the graph structure and node attribute information to obtain a better node representation that preserves both the topological structure and node attribute similarity. Therefore, with the two decoders, the GAEPNAS model can implement unsupervised learning by comparing the adjacency matrix and attribute similarity matrix of the embedded space with those of the original space, so that obtaining a noderepresentation can effectively preserve both the topological structure and node attribute similarity.

### 3.5. Loss Function

In the GAEPNAS model, the primary focus is on the error that arises between the reconstructed adjacency matrix and that of the original graph. Additionally, the error between the reconstructed similarity matrix and the node attribute matrix of the original graph is also taken into account. Thus, a structure loss and a similarity loss are defined to measure the two errors. The structure decoder reconstructs the adjacency matrix $A^{\prime }$ by computing the inner product. As the input adjacency matrix A is a 0/1 matrix, the structure loss $L_{str}$ is defined using a cross-entropy of the following form:

(10) $$\begin{array}{c} L_{str}=-\frac{1}{n^{2}}\sum_{i=1}^{n}\sum_{j=1}^{n}\left(A_{ij}logA_{ij}^{\prime }+(1-A_{ij})log(1-A_{ij}^{\prime })\right) \end{array}$$

The similarity decoder reconstructs the attribute similarity matrix. The similarity loss is defined as

(11) $$\begin{array}{c} L_{sim}={\frac{1}{n^{2}}\left\|S-S^{\prime }\right\|}_{F}^{2}=\frac{1}{n^{2}}\sum_{i=1}^{n}\sum_{j=1}^{n}{\left(S_{ij}-S_{ij}^{\prime }\right)}^{2} \end{array}$$

where ${\left\|.\right\|}_{F}$ is the Frobenius norm.

Therefore, the overall loss function is expressed as

(12) $$\begin{array}{c} {L=\lambda L}_{str}+(1-\lambda )L_{sim} \end{array}$$

where λ∈[0,1] is a parameter used to balance the structure loss and the similarity loss.

### 3.6. Training Algorithm

The whole GAEPNAS model is optimized by minimizing the loss function L. The training algorithm for GAEPNAS is presented in Algorithm 2. Given an attribute network G=(V,E,X), Algorithm 1 is initially called to convert the neighborhood relationship of the nodes in the attribute space into a KNN graph based on their attribute similarity, and then fuse it with the topology graph of the original network so that the fused graph has both the topology of the original network and the neighborhood relationship of the nodes’ attributes (line 1). The self-loops are then added to the fusion graph, the degree matrix is computed, and the parameters of the GAEPNAS model are initialized in preparation for the model training (lines 2–4). Subsequently, t-rounds are started to train the model (lines 5–14). In the forward propagation process (lines 6–12), the node representation matrix Z is obtained by l-layers GCN of the encoder (lines 6–9), the adjacency matrix and similarity matrix are reconstructed, and the loss function is calculated (lines 10–12). The model is updated with its stochastic gradient by minimizing the loss function (line 13). After t rounds of the model training, the graph embedding Z is derived and a trained model is ultimately obtained.

The time complexity of the training algorithm is analyzed in the sequel. It can be seen that the time complexity of Algorithm 1 to compute matrices S and $A_{F}$ is $O({cn}^{2})$, where n is the number of nodes and c is the dimension of the node attributes. In lines 2–4, the time complexity of some simple addition and initialization operations is O(1). In lines 5–14, the reconstruction of the node attribute similarity matrix is added to the general GAE model to train the proposed GAEPNAS. Thus, the main calculation amount is attributed to the training of the model and the computing of the similarity matrix $S^{\prime }$. The time complexity of the general GAE model is $O(tmcd_{1}{\cdots d}_{l})$ and that of the computation of matrix $S^{\prime }$ is $O({d_{l}n}^{2})$, where t denotes the number of iterations, m denotes the number of edges, and $d_{i}(i=1,2,\cdots ,l)$ denotes the dimension of the i-layer of the encoder. In summary, the time complexity of the model training is $O(tmcd_{1}{\cdots d}_{l}+{cn}^{2}+{d_{l}n}^{2})$.
**Algorithm 2.** Training GAEPNAS**Input:** Adjacency matrix A, Attribute matrix X, Number of nearest neighbors k, Number of iterations t, Number of hidden layers l, and Parameters β and λ.**Output:** Node Representation matrix Z and model parameter $W=\{W^{\left(0\right)},W^{\left(1\right)},\ldots ,W^{\left(l-1\right)}\}$.1.Compute similarity matrix S and adjacency matrix $A_{F}$ of graph $G_{F}$ by calling Algorithm 1;2.Compute $\tilde{A}=I_{n}+A_{F}$ and diagonal matrix $\tilde{D}$ with ${\tilde{D}}_{ii}=\sum_{j=1}^{n}{\tilde{A}}_{ij}$;3.Initialize model parameter $W=\{W^{\left(0\right)},W^{\left(1\right)},\ldots ,W^{\left(l-1\right)}\}$;4.$H^{\left(0\right)}=X$;5.**for** i=1:t6.   **for** j=0:(l−1)7.      $H^{\left(j+1\right)}=\sigma \left({{\tilde{D}}^{-\frac{1}{2}}\tilde{A}{\tilde{D}}^{-\frac{1}{2}}H}^{\left(j\right)}W^{\left(j\right)}\right)$;8.   **end**
9.   $Z=H^{\left(l\right)}$;10.   Reconstruct adjacency matrix $A^{\prime }=sigmoid\left(ZZ^{T}\right)$;11.   Reconstruct similarity matrix $S^{\prime }$ by specific similarity function;12.   Compute ${L=L}_{str}+\lambda L_{sim}$ according to Equations (10) and (11);13.Compute partial derivative $\frac{\partial L}{\partial W^{\left(i\right)}}$ with back-propagation algorithm to update model parameter $W=\{W^{\left(0\right)},W^{\left(1\right)},\ldots ,W^{\left(l-1\right)}\}$;14.**end**15.**return** Z and W;

## 4. Experiments

In this section, experiments are conducted on benchmark datasets, and the effectiveness of the proposed GAEPNAS is evaluated with relevant baselines on two classic downstream tasks, namely link prediction and node clustering. The experimental environment is as follows:

Intel(R) Core(TM) i7-12700 CPU @2.10 GHz 4.90 GHz, 128 G of RAM, NVIDIA Ge-Force RTX 3090 GPU; Ubuntu22.04.1LTS, Python 3.6.13, Pytorch 1.10.2.

### 4.1. Datasets

To evaluate the effectiveness of the proposed model, three public real-world citation network datasets (Cora, CiteSeer, and PubMed), which are the most commonly used graph datasets to evaluate the GAE and VGAE models, are considered. In these citation networks, the nodes represent scientific papers and the links represent citation relationships between papers. Cora’s papers are from machine learning fields, and they are classified into seven classes; CiteSeer contains papers from six categories in the computer science field; and PubMed contains scientific papers related to diabetes and its papers can be classified into three classes. The detailed information is presented in Table 1.

In the Cora and Citseer datasets, the attribute vector of each paper takes the form of 0/1-valued word vector indicating the absence/presence of the corresponding word from the dictionary. When a word does not present in either paper, it does not impact the similarity between the two papers. Thus, in the original attribute space, the attribute similarity between attribute vectors $X_{i}$ and $X_{j}$ can be expressed as

(13) $$\begin{array}{c} S_{ij}=Sim\left(X_{i},X_{j}\right)=\frac{\sum_{h=1}^{c}\left|X_{ih}\wedge X_{jh}\right|}{\sum_{h=1}^{c}\left(\left|X_{ih}\wedge X_{jh}\right|+\left|X_{ih}⨁X_{jh}\right|\right)} \end{array}$$

where ∧ and ⨁ respectively denote the “AND” and “XOR” operations and c is the dimension of the node attributes.

In the PubMed dataset, the attribute vector of each paper contains term frequency–inverse document frequency (TF–IDF) scores for 500 words. The attribute similarity between nodes is calculated by the cosine similarity in the original attribute space because the attribute vectors have continuous real values.

In the embedding space of nodes, all three use cosine similarity to calculate the attribute similarity between nodes because their node representations have continuous real values.

### 4.2. Baseline Methods

Some classical baseline methods are considered for comparison with the proposed GAEPNAS. In most related studies, these methods have been used for comparison on link prediction and node clustering tasks. These methods are summarized as follows:

**Spectral** clustering [38]: an effective graph representation method to perform dimensionality reduction by the eigenvalues.

**DeepWalk** [6]: a classical graph representation method that trains the skip-gram model for sequences generated by random walk on graphs.

**GAE** and **VGAE** [12]: popular unsupervised graph representation methods that combine GCN with the (variational) autoencoder to graph representation learning.

**Graphite-AE** and **Graphite-VAE** [39]: variants of the GAE and VGAE methods that reconstruct the original graph by a multilayer iterative procedure.

**ARGA** and **ARVGA** [17]: variants of the GAE and VGAE methods that use adversarial models to learn node representation.

**Linear GAE** and **Linear VGAE** [40]: simplified versions of the GAE and VGAE methods with one-hop linear models.

**GASN** [35]: a variant of the GAE method where the graph structure and node attribute are reconstructed using two decoders.

**AT-GAE** and **AT-VGAE** [41]: generalization methods of GAE and VGAE using adversarial training.

### 4.3. Link Prediction

Link prediction is one of the most important tasks in graph analysis. Its objective is to find missing links between nodes or predict possible links between nodes in the future. The performance of GAEPNAS is evaluated with the link prediction task. In the experiments, the adjacency matrix reconstructed by the decoder is directly used as the predicted adjacency matrix.

#### 4.3.1. Metrics of Link Prediction

The accuracy of various link prediction methods is determined using two metrics: the area under a receiver operating characteristic curve (AUC) score and the average precision (AP) score. The AUC is defined as the area enclosed by the coordinate axis under the receiver operating characteristic (ROC). It is used to quantify the accuracy of link prediction. In practice, it can be seen as the probability that the link prediction score of a missing link randomly chosen is higher than a nonexistent link [42]. For n independent comparisons, if missing links having a higher score exist $n^{\prime }$ times and those having the same score exist $n^{\prime \prime }$ times, then the AUC is calculated as:

(14) $$\begin{array}{c} AUC=\frac{n^{\prime }+0.5n^{\prime \prime }}{n} \end{array}$$

Because the ROC curve is located above the line y=x and its area is not greater than 1, AUC∈[0.5,1]. For a link prediction algorithm, the closer the AUC to 1, the higher its accuracy.

The AP quantifies the precision of link prediction, which is defined as the area under the precision–recall curve. In practice, it is calculated by the weighted mean of precisions achieved at each threshold, while the increase in recall from the previous threshold is used as the weight [43]:

(15) $$\begin{array}{c} AP=\sum_{k}\left(R_{k}-R_{k-1}\right)P_{k} \end{array}$$

where $R_{k}$ and $P_{k}$ are the recall and precision at the k-th threshold, which are calculated as in [43]. For a link prediction algorithm, the higher the AP value, the higher its precision.

#### 4.3.2. Implementation

For each dataset, all the edges are randomly split into a training set (85%), a validation set (5%), and a test set (10%). In addition, the same number of non-edges is randomly sampled as negative samples added to both the validation set and the test set. Because the Cora and CiteSeer are relatively small datasets, the GAEPNAS was trained for 200 epochs and updated using the Adam algorithm with a learning rate of 0.001. For PubMed, the model is trained for 500 epochs and optimized using the Adam algorithm with a learning rate of 0.01. The parameter settings of the GAEPNAS model are shown in Table 2. Because the characteristics of each dataset (e.g., the average degree of nodes, the dimensionality and distribution of attributes, etc.) are different, the values k, β and λ in Table 2 are also taken differently. All parameters of the baseline methods are set according to their original papers. Each experiment is randomly run 10 times, and the averaged AUC and AP are determined.

#### 4.3.3. Experimental Results

The experimental results of link prediction are shown in Table 3, where the best results are marked in bold. Note that the inputs of Spectral and DeepWalk include only graph structure, while the inputs of the other methods include graph structure and node attributes. The GASN and GAEPNAS methods aim to reconstruct the node attributes and attribute similarity, while the other methods (GAE and VGAE, Graphite AE and VAE, ARGA and ARVGA, Linear GAE and VGAE) only focus on reconstructing the graph structure. It can be observed from Table 2 that the methods using graph structure and node attribute information perform significantly better than those (Spectral and DeepWalk) only using graph structure information. This shows that both the graph structure and node attribute information are beneficial for the link prediction task. In addition, the GASN and GAEPNAS methods, which reconstruct both the graph structure and the node attribute information, perform better than the other methods that only reconstruct graph structure information. This is because the preservation of node attribute information is crucial. Furthermore, GAEPNAS performs better than GASN for the Cora and CiteSeer datasets, but slightly worse than GASN for the PubMed dataset. The proposed GAEPNAS model integrates the graph structure information with the node attribute similarity information in the encoder by the KNN graph, and reconstructs the graph structure and node attribute similarity in the decoder, while the GASN model uses a general encoder and reconstructs the graph structure and node attribute in the decoder. Thus, the proposed model can more adequately learn the node attribute similarity information to node representation in the encoder, resulting in higher link prediction performance.

#### 4.3.4. Parameter Analysis

**Figure 2:**
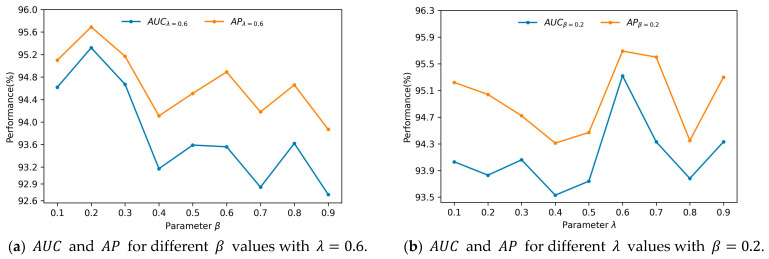
Link prediction performance of GAEPNAS for different *β* and *λ* values on the Cora dataset.

**Figure 3:**
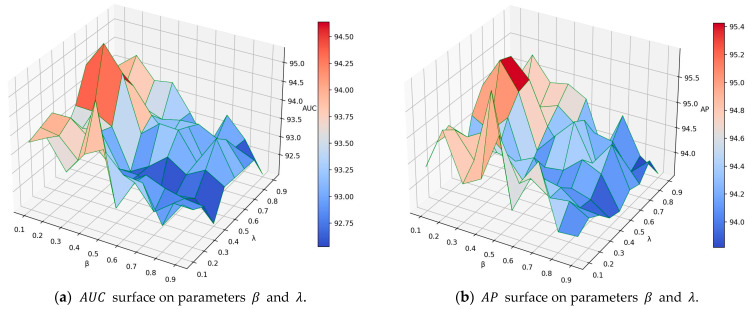
Impact of parameters *β* and *λ* on the link prediction performance of GAEPNAS on the Cora dataset.

The main parameters that affect the performance of GAEPNAS are β in Equation (4) and λ in Equation (12). This paper only studies the impact of these two parameters on the link prediction task for the Cora dataset. In Equation (4), as β increases, the ability of the model to learn graph structural information becomes more powerful while the ability to learn the node attribute similarity becomes weaker. In Equation (12), as λ increases, it is expected that more information about graph structure is preserved in node representation and less information about node attribute similarity is preserved accordingly. In the experiments, β and λ are chosen in the range of [0.1,0.9] with an interval of 0.1. The model achieves the highest performance for β=0.2 and λ=0.6. By fixing λ to 0.6, the AUC and AP curves of parameter β are plotted (Figure 2a). Similarly, by fixing β to 0.2, the AUC and AP curves of parameter λ are drawn (Figure 2b). The 3D surface plots of AUC and AP for β and λ are shown in Figure 3a,b. It can be observed from Figure 2a that as β increases, AUC and AP first increase and then decrease. When β=0.2, AUC and AP reach their maximum values. This may be due to the fact that the link prediction for the Cora dataset is mainly based on the similarity between node attributes, while relying on less information about the graph structure. From Figure 2b, it can be observed that as λ increases, AUC and AP first decrease, then increase, and finally decrease again. When λ = 0.6, AUC and AP reach their maximum values. Moreover, when λ increases into the [0.6,0.7] interval, the two types of information preserved in the node representation reach a relative balance, which results in a higher link prediction performance. From Figure 3 it can be observed that as β increases, AUC and AP first increase and then decrease. Furthermore, the GAEPNAS model achieves a high performance of link prediction when β∈[0.1,0.3] and λ∈[0.6,0.7]. The AUC and AP vary with parameters β and λ in the ranges of [0.921,0.953] and [0.936,0.960], respectively. Thus, the GAEPNAS model can lead to acceptable results for all the combinations of parameters β and λ.

### 4.4. Node Clustering

Node clustering is one of the fundamental unsupervised methods of graph analysis. Its goal is to classify similar nodes into the same clusters and dissimilar nodes in different clusters without supervision or prior knowledge of the clusters. In the experiments, node clustering is used as a downstream task to evaluate GAEPNAS.

#### 4.4.1. Metrics of Node Clustering

The clustering results are measured using three metrics: clustering accuracy (ACC), normalized mutual information (NMI), and adjusted Rand index (ARI). The values of these metrics are in the range of [0,1], while the larger the value, the higher the clustering performance. It is assumed that $C=\{C_{1},C_{2},\ldots ,C_{k}\}$ and $C^{\prime }=\{C_{1}^{\prime },C_{2}^{\prime },\ldots ,C_{k^{\prime }}^{\prime }\}$ are, respectively, the ground truth classes and the clustering results of the dataset with n nodes, where $C_{1}$ and $C_{1}^{\prime }$ represent a ground-truth class and a cluster, respectively. Let $n_{ij}=|C_{i}^{\prime }⋂C_{j}|$, $n_{C_{i}}=|C_{i}|$ and $n_{C_{i}^{\prime }}=|C_{i}^{\prime }|$; the three metrics are then defined as follows:

The *ACC* measures the percentage of the best matching between the clustering result and the ground-truth class:

(16) $$\begin{array}{c} ACC=\frac{\sum_{i=1}^{k^{\prime }}{max}_{j=1}^{k}n_{ij}}{n} \end{array}$$

The *NMI* measures the normalized similarity between the predicted clusters and the ground truth classes [44]:

(17) $$\begin{array}{c} NMI=-\frac{\sum_{i=1}^{k^{\prime }}\sum_{j=1}^{k}n_{ij}\frac{n∙n_{ij}}{n_{C_{i}^{\prime }}∙n_{C_{j}}}}{\frac{1}{2}\left(\sum_{j=1}^{k}n_{C_{j}}log\frac{n_{C_{j}}}{n}+\sum_{i=1}^{k}n_{C_{i}^{\prime }}log\frac{n_{C_{i}^{\prime }}}{n}\right)} \end{array}$$

where the numerator is the mutual information between the ground truth classes C and the predicted clusters $C^{\prime }$ and the denominator is the arithmetic mean for the information entropy between C and $C^{\prime }$. If C=C′, NMI=1; and if C and $C^{\prime }$ are completely different, NMI=0.

The *ARI* measures the similarity between the predicted clusters and the ground truth classes based on the pairwise comparison of included nodes [45]:

(18) $$\begin{array}{c} ARI=\frac{\left(\begin{array}{c} n\\ 2 \end{array}\right)\sum_{i=1}^{k^{\prime }}\sum_{j=1}^{k}\left(\begin{array}{c} n_{ij}\\ 2 \end{array}\right)-\sum_{i=1}^{k^{\prime }}\left(\begin{array}{c} n_{C_{i}^{\prime }}\\ 2 \end{array}\right)\sum_{j=1}^{k}\left(\begin{array}{c} n_{C_{j}}\\ 2 \end{array}\right)}{\frac{1}{2}\left(\begin{array}{c} n\\ 2 \end{array}\right)\left(\sum_{i=1}^{k^{\prime }}\left(\begin{array}{c} n_{C_{i}^{\prime }}\\ 2 \end{array}\right)+\sum_{j=1}^{k}\left(\begin{array}{c} n_{C_{j}}\\ 2 \end{array}\right)\right)-\sum_{i=1}^{k^{\prime }}\left(\begin{array}{c} n_{C_{i}^{\prime }}\\ 2 \end{array}\right)\sum_{j=1}^{k}\left(\begin{array}{c} n_{C_{j}}\\ 2 \end{array}\right)} \end{array}$$

#### 4.4.2. Implementation

In the experiments, node representations are first obtained by the baseline methods and the proposed method, and then clustered into classes using the K-Means clustering algorithm. The cluster number in K-Means is set as the number of classes for each dataset. For the node clustering task, we do not use any label in the unsupervised learning process. As with the link prediction task, the dataset is randomly separated into a training set (85% edges), a validation set (5% edges), and a test set (10% edges) for each experiment. The experimental parameters of GAEPNAS are set as shown in Table 4. All parameters of the baseline methods are set according to their original papers. Each experiment is also repeated 10 times, and the averaged *ACC*, *NMI*, and *ARI* are determined.

#### 4.4.3. Experimental Results

The experimental results of the node clustering task are presented in Table 5. It can be observed that the models that use both structure and attribute information perform significantly better than Spectral and DeepWalk, which solely rely on graph structure information. Thus, the node attribute information plays a crucial role in enhancing the node clustering performance. The proposed GAEPNAS model shows superior performance compared to GAE-based models (GAE and VGAE, Graphite AE and VAE, ARGA and ARVGA, Linear GAE and VGAE) that only reconstruct the graph structure. Specifically, the GAEPNAS model demonstrates better performance than the GASN, which reconstructs both the graph structure and node attribute. GAEPNAS incorporates the structural and attribute similarity information in the encoder and simultaneously reconstructs the graph structure and node attribute similarity. Furthermore, the effective integration and complementarity of both types of information improve its node clustering performance. Thus, the GAEPNAS model outperforms the other models on all three datasets, as shown in the results of all the metrics.

#### 4.4.4. Parameter Analysis

**Figure 4:**
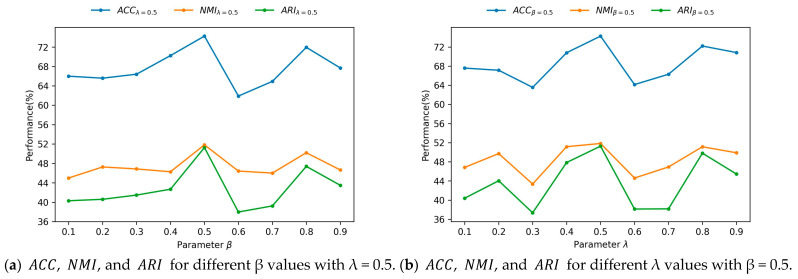
Node clustering performance of GAEPNAS for different values of β and λ on the Cora dataset.

**Figure 5:**
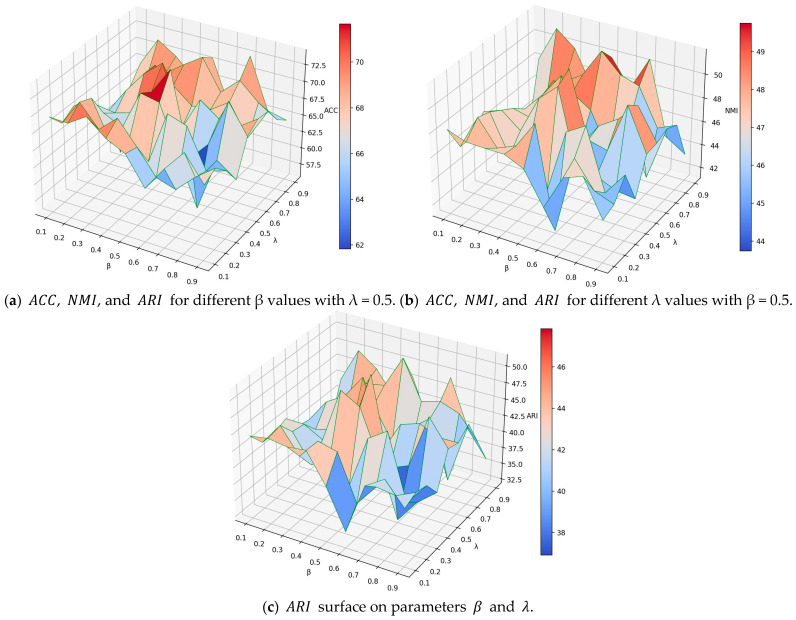
Impact of *β* and *λ* on the node clustering performance of GAEPNAS on the Cora dataset.

In this subsection, the impact of parameters β and λ on the node clustering results is studied for the Cora dataset. Similarly to the previous link prediction task, β and λ are varied in the range of [0.1,0.9] with an interval of 0.1. The model shows the highest node clustering performance for β=0.5 and λ=0.5. The ACC, NMI, and ARI curves of β with λ=0.5 are shown in Figure 4a. Similarly, the ACC, NMI, and ARI curves of λ with β=0.5 are shown in Figure 4b. The 3D surface plots of ACC, NMI, and ARI for β and λ are shown in Figure 5a–c, respectively. It can be observed from Figure 4a,b that the performance measured by ACC, NMI, and ARI is low at both ends and high in the middle. The maximum values of ACC, NMI, and ARI are achieved for β=0.5 and λ=0.5, respectively. Figure 5 shows that the GAEPNAS model achieves high clustering performance for β∈[0.4,0.6] and λ∈[0.5,0.7]. In node clustering, similar nodes are classified into the same clusters and dissimilar nodes are grouped in different clusters. Nodes in the same cluster are expected to be adjacent to each other and similar in attribute space; while nodes in different clusters are dissimilar and not adjacent. Therefore, the node attribute similarity information plays the same role in node clustering as graph structural information. As a result, β and λ should be reasonable and interpretable in the range of [0.4,0.6] and [0.5,0.7], respectively. As can be observed in Figure 4 and Figure 5, the performance of the proposed model is slightly sensitive to the parameters β and λ.

### 4.5. Ablation Study

To further analyze the effectiveness of the various modules in the proposed model, an ablation study on the Cora and CiteSeer datasets is conducted in four different configurations: **GAE** (the general GAE model), **GAE+KNN** (adding the fusion module to the general GAE model), **GAE+RecSim** (adding a node attribute similarity decoder and corresponding reconstruction loss to the general GAE model), and **GAEPSNA** (GAE+ KNN+Recsim).

The ablation experimental results are presented in Table 6 and Table 7. It can be observed that GAE has the worst results because it only preserves the structural information in the learning process. Additionally, GAE+KNN and GAE+Recsim show higher performance than GAE due to their strengthened learning of node attribute information. Furthermore, the proposed GAEPNAS model has the highest performance. This is because its learning process enhances the learning for node attribute information and emphasizes the preservation of node attribute similarity in the node representation. It can also be deduced that each module of the proposed model is effective and can contribute to its high performance.

## 5. Discussion

The GAEPNAS model fuses the KNN graph that can represent the attribute similarity between nodes with the original graph, then learns the representation of the nodes from the synthetic graph though a GCN encoder, and finally reconstructs the original graph structure and attribute similarity between nodes by leveraging dual decoders so that the nodes maintain the same attribute similarity between nodes in the embedding space as in the original space. Despite the model’s effectiveness, there are some limitations. Below, we discuss two major limitations of the model and how they can be overcome in future works.

(1) Computational scalability: In the proposed model, we need to calculate the node attribute similarity for each pair of nodes in the graph in order to construct the KNN graph and reconstruct the node attribute similarity matrix, which results in quadratic computational complexity. Thus, for real-world large graphs, the theoretical complexity is unacceptable. However, in real networks, it is difficult for two nodes to interact with each other if they are not in the same connected component, even if they are similar in node attributes. The closer they are in the network, the more interconnected they are. For example, in a citation network, researchers usually search papers with no more than five hop neighbors to keep track of a paper. Therefore, in the future, in order to reduce computational overhead, we will consider computing the attribute similarity between each node and its k-hop neighbors to avoid computing the full pairwise similarity matrix.

(2) Fusion of structural and attribute similarity information: It was experimentally discovered that our model’s performance is slightly sensitive to the parameters β and λ. β is used to balance the fusion effect of the original input graph and the KNN graph, and λ is used to balance the structure loss and the similarity loss. For the performance of the proposed model, the balance of structural and attribute similarity information is crucial. However, in Equation (4), β is a scalar parameter which is difficult to adapt to the requirements of complex networks. Setting the parameter β as a learnable vector or matrix may be an effective way to do this. Therefore, in future work, we will attempt to introduce learnable parameters β and λ into the GAEPNAS model to reach more stable performance.

## 6. Conclusions

In this paper, a novel GAE model with preserving node attribute similarity is proposed for unsupervised graph representation learning. In this model, graph structural information and node attribute information are fused by generating a KNN graph. The model then learns the fusion information by sharing the same set of parameter weights and using two different aggregating methods in the encoder. In the decoder module, the model uses two different decoders to simultaneously reconstruct the graph structure information and node attribute similarity information. The structure loss and attribute similarity loss are used to update the parameters and preserve the graph structure information and node attribute similarity information in node representations. The experimental results show that the proposed model outperforms baseline methods in link prediction and node clustering on three citation networks. Finally, we discuss two limitations of the GAEPNAS model and provide an outlook for future research.

## Figures and Tables

**Table 1:** Datasets information.

Dataset	# Nodes	# Edges	# Attributes	Attribute Type	# Classes
Cora	2708	5429	1433	Binary	7
CiteSeer	3327	4732	3703	Binary	6
PubMed	19,717	44,338	500	Continuous	3

**Table 2:** Experimental settings for training the GAEPNAS model in the link prediction task.

Dataset	Epochs	Learning Rate	# Neurons	*k*	*β*	*λ*
Cora	200	0.001	1433-512-16	45	0.2	0.6
CiteSeer	200	0.001	3703-128-32	35	0.1	0.4
PubMed	500	0.01	500-256-16	45	0.8	0.4

**Table 3:** Experimental results (%) in the link prediction task.

Method	Cora		CiteSeer		PubMed	
	*AUC*	*AP*	*AUC*	*AP*	*AUC*	*AP*
Spectral	84.6	88.5	80.5	85.0	84.2	87.8
DeepWalk	83.1	85.0	80.5	83.6	84.4	84.1
GAE	91.0	92.0	89.5	89.9	96.4	96.5
VGAE	91.4	92.6	90.8	92.0	94.4	94.7
Graphite-AE	91.0	92.8	92.6	94.1	94.5	95.7
Graphite-VAE	91.5	93.2	93.5	95.0	94.6	96.0
ARGA	92.4	93.2	91.9	93.0	96.8	97.1
ARVGA	92.4	92.6	92.4	93.0	96.5	96.8
Linear GAE	92.1	93.3	91.5	93.0	95.9	95.9
Linear VGAE	92.6	93.7	91.6	93.1	95.9	95.8
GASN	93.8	94.2	93.5	95.1	**96.8**	**97.2**
GAEPNAS	**95.3**	**95.7**	**96.3**	**96.6**	96.7	97.0

**Table 4:** Experimental settings for training the GAEPNAS model in the node clustering task.

Dataset	Epochs	Learning Rate	# Neurons	*k*	*β*	*λ*
Cora	200	0.001	1433-512-16	7	0.5	0.5
CiteSeer	200	0.001	3703-128-32	15	0.5	0.4
PubMed	500	0.01	500-256-16	15	0.4	0.6

**Table 5:** Experimental results (%) on the node clustering task.

Method	Cora			CiteSeer			PubMed		
	*ACC*	*NMI*	*ARI*	*ACC*	*NMI*	*ARI*	*ACC*	*NMI*	*ARI*
Spectral	36.7	12.6	3.1	23.8	5.5	1.0	52.8	9.7	6.2
DeepWalk	48.4	32.7	24.2	33.6	8.7	9.2	68.4	27.9	29.9
GAE	59.6	42.9	34.7	40.8	17.6	12.4	67.2	27.7	27.9
VGAE	50.2	32.9	25.4	46.7	26.0	20.5	63.0	22.9	21.3
ARGA	64.0	44.9	35.2	57.3	35.0	34.1	66.8	30.5	29.5
ARVGA	63.8	45.0	37.4	54.4	26.1	24.5	69.0	29.0	30.6
AT-GAE	67.1	51.4	43.4	61.6	36.3	34.6	68.4	31.9	30.2
AT-VGAE	67.3	50.5	44.3	60.4	36.5	34.7	69.8	33.2	32.5
GASN	66.9	48.4	39.2	60.3	38.6	37.1	69.2	31.3	31.0
GAEPNAS	**74.3**	**51.8**	**51.3**	**66.1**	**39.2**	**40.2**	**71.3**	**34.5**	**35.2**

**Table 6:** Ablation experimental results of link prediction.

Method	Cora		CiteSeer	
	*AUC*	*AP*	*AUC*	*AP*
GAE	91.0	92.0	89.5	89.9
GAE+KNN	93.2	93.9	94.6	95.2
GAE+RecSim	93.1	94.2	93.8	94.5
GAEPNAS	**95.3**	**95.7**	**96.5**	**96.6**

**Table 7:** Ablation experimental results of node clustering.

Method	Cora			CiteSeer		
	*ACC*	*NMI*	*ARI*	*ACC*	*NMI*	*ARI*
GAE	59.6	42.9	34.7	40.8	17.6	12.4
GAE+KNN	71.2	50.2	50.1	62.1	34.1	35.3
GAE+RecSim	69.9	49.8	48.2	60.4	35.7	33.9
GAEPNAS	**74.3**	**52.2**	**51.6**	**63.2**	**37.8**	**37. 0**

## Data Availability

Not applicable.
